# Macular vessel density in the superficial plexus is not a proxy of cerebrovascular damage in non-demented individuals: data from the NORFACE cohort

**DOI:** 10.1186/s13195-024-01408-9

**Published:** 2024-02-20

**Authors:** Ainhoa García-Sánchez, Oscar Sotolongo-Grau, Juan Pablo Tartari, Ángela Sanabria, Ester Esteban - De Antonio, Alba Pérez-Cordón, Montserrat Alegret, Vanesa Pytel, Joan Martínez, Núria Aguilera, Itziar de Rojas, Amanda Cano, Pablo García-González, Raquel Puerta, Clàudia Olivé, Maria Capdevila, Fernando García-Gutiérrez, Assumpta Vivas, Marta Gómez-Chiari, Juan Giménez, Miguel Ángel Tejero, Miguel Castilla-Martí, Luis Castilla-Martí, Lluís Tárraga, Sergi Valero, Agustín Ruiz, Mercè Boada, Marta Marquié, on behalf of the FACEHBI study group, J. A. Alllué, F. Appiani, D. M. Ariton, M. Berthier, U. Bojayrin, M. Buendia, S. Bullich, F. Campos, S. Castillo, P. Cañabate, L. Cañada, C. Cuevas, S. Diego, A. Espinosa, A. Gailhajenet, M. Guitart, M. Ibarria, A. Lafuente, N. Lleonart, F. Lomeña, E. Martín, M. Moreno, A. Morera, L. Montrreal, A. Niñerola, A. B. Nogales, L. Núñez, A. Orellana, G. Ortega, A. Páez, A. Pancho, E. Pelejà, E. Pérez-Martínez, V. Pérez-Grijalba, M. Pascual-Lucas, A. Perissinotti, S. Preckler, M. Ricciardi, N. Roé-Vellvé, J. Romero, M. I. Ramis, M. Rosende-Roca, M. Sarasa, S. Seguer, A. W. Stephens, J. Terencio, M. Torres, L. Vargas, on behalf of the BIOFACE study group, F. Appiani, D. M. Ariton, U. Bojayrin, M. Buendía, A. Calvet, M. J. Castillón, P. Cañabate, L. Cañada, C. Cuevas, I. de Rojas, S. Diego, A. Espinosa, A. Gailhajenet, M. Guitart, M. Ibarria, A. Lafuente, N. Lleonart, E. Martín, M. Moreno, A. Morera, L. Montrreal, A. B. Nogales, A. Orellana, G. Ortega, A. Pancho, S. Preckler, M. Ricciardi, M. I. Ramis, M. Rosende-Roca, S. Seguer, L. Vargas

**Affiliations:** 1 Ace Alzheimer Center Barcelona, Universitat Internacional de Catalunya (UIC), Barcelona, Spain; 2 CIBERNED, Center for Networked Biomedical Research On Neurodegenerative Diseases, National Institute of Health Carlos III, Madrid, Spain; 3 Department of Diagnostic Imaging, Clínica Corachan, Barcelona, Spain; 4 Clínica Oftalmológica Dr. Castilla, Barcelona, Spain; 5 Vista Alpina Eye Clinic, Visp, Switzerland; 6 PhD Programme in Surgery and Morphological Sciences, Universitat Autònoma de Barcelona, Barcelona, Spain; 7 Hôpital Ophtalmique Jules-Gonin, Fondation Asiles Des Aveugles, University of Lausanne, Lausanne, Switzerland

**Keywords:** Vessel density, Optical coherence tomography-angiography, Cerebrovascular damage, Brain atrophy, NORFACE, FACEHBI, BIOFACE

## Abstract

**Introduction:**

Optical coherence tomography angiography (OCT-A) is a novel tool that allows the detection of retinal vascular changes. We investigated the association of macular vessel density (VD) in the superficial plexus assessed by OCT-A with measures of cerebrovascular pathology and atrophy quantified by brain magnetic resonance imaging (MRI) in non-demented individuals.

**Methods:**

Clinical, demographical, OCT-A, and brain MRI data from non-demented research participants were included. We analyzed the association of regional macular VD with brain vascular burden using the Fazekas scale assessed in a logistic regression analysis, and the volume of white matter hyperintensities (WMH) assessed in a multiple linear regression analysis. We also explored the associations of macular VD with hippocampal volume, ventricle volume and Alzheimer disease cortical signature (ADCS) thickness assessed in multiple linear regression analyses. All analyses were adjusted for age, sex, syndromic diagnosis and cardiovascular variables.

**Results:**

The study cohort comprised 188 participants: 89 with subjective cognitive decline and 99 with mild cognitive impairment. No significant association of regional macular VD with the Fazekas categories (all, *p* > 0.111) and WMH volume (all, *p* > 0.051) were detected. VD in the nasal quadrant was associated to hippocampal volume (*p* = 0.007), but no other associations of macular VD with brain atrophy measures were detected (all, *p* > 0.05).

**Discussion:**

Retinal vascular measures were not a proxy of cerebrovascular damage in non-demented individuals, while VD in the nasal quadrant was associated with hippocampal atrophy independently of the amyloid status.

**Supplementary Information:**

The online version contains supplementary material available at 10.1186/s13195-024-01408-9.

## Introduction

Cerebrovascular (CV) damage is a very common concomitant pathology to Alzheimer disease (AD) in the elderly, as shown in autopsy studies [1, 2] and is also involved in the pathophysiology of cognitive impairment [3]. In fact, the most frequent underlying cause of dementia in the elderly is mixed pathology (co-existence of AD and CV damage) [4]. Also, well-established cardiovascular risk factors such as high blood pressure, diabetes mellitus, smoking, and obesity are associated with an increased risk of developing AD [5].

The brain shares with the retina several developmental, functional, and pathophysiological features. Both are connected through bundles of neuronal axons forming the optic nerve and also blood vessels [6]. Related to the latter, the inner blood-retinal barrier is an analog of the blood–brain barrier [7]. Retinal capillary density differs regionally, in parallel to that in the brain, being greatest in the macula, while the periphery of the retina is almost avascular [8]. The retinal capillary network is organized in two distinct beds: the superficial plexus at the level of the ganglion cell layer, and the deep plexus at the outer plexiform layer [9].

The retinal microvasculature can be directly assessed in vivo using Optical Coherence Tomography Angiography (OCT-A), while this is not possible for brain vessels through brain magnetic resonance imaging (MRI), thus the retina is considered to be “a window into the brain”. CV pathology usually remains undetected until significant damage has occurred and causes symptoms that warrant performance of brain imaging, while retinal vasculopathy may be identified non-invasively by OCT-A imaging early in the disease process. OCT-A obtains high-resolution images of the retina based on backscattered light from its neurosensory and vascular tissues, and it allows the visualization of retinal vascular abnormalities such as microaneurysms, neovascularization, vascular non-perfusion, reduced vascular density (VD), and enlarged foveal avascular zone (FAZ) [10]. OCT-A obtained the U.S. Food and Drug Administration (FDA) approval in 2016 and in these past few years it has been used to evaluate a spectrum of ocular vascular diseases including diabetic retinopathy, retinal venous occlusion, uveitis, retinal arterial occlusion, and age-related macular degeneration, among others [10]. Growing evidence indicates that microvascular retinal changes could be markers of CV, neurodegenerative, and psychiatric diseases as well. In particular, in the field of cognitive impairment, changes in retinal vascular network geometry have been correlated with worse cognitive functioning [11], and several retinopathy signs have been associated with vascular and AD dementia [11, 12]. Also, several OCT-A quantitative retinal measures, such as VD and the size of the FAZ, have been investigated, pointing to a retinal vascular loss in mild cognitive impairment (MCI) and AD dementia patients compared to healthy controls [13–19].

Thus, OCT-A is an emerging area of research in the field of novel biomarkers for cognitive decline and offers an exceptional opportunity to assess non-invasively both the retinal and also the brain microvasculatures.

In the present study, we explored OCT-A and brain MRI data from 188 non-demented participants from the Neuro-ophthalmology Research at Fundació ACE (NORFACE) cohort. Our main goal was to assess whether there was an association between retinal vascular damage (quantified as macular VD in the superficial plexus by OCT-A) and brain vascular damage (quantified as the Fazekas scale and the volume of white matter hyperintensities (WMH) by brain MRI). Additionally, we aimed to assess (1) the association between macular VD and several brain atrophy measures and (2) the influence of the amyloid status (+ / −) measured by positron emission tomography (PET) in the association of retinal vascular damage with brain vascular and atrophy measures. Our hypothesis was that retinal vascular measures are a proxy of CV damage and thus OCT-A could be used a surrogate marker of CV pathology, regardless of the presence of brain amyloidosis.

## Materials and methods

### Study subjects

The Neuro-Ophthalmology Research at Fundació ACE (NORFACE) cohort was founded in 2014 to investigate retinal biomarkers of AD and examine the relationship between retinal changes and different types of neurodegenerative disorders [20]. In the present study, we included non-demented participants from the FACEHBI [21] and BIOFACE [22] research cohorts at Ace Alzheimer Center Barcelona with a diagnosis of either Subjective Cognitive Decline (SCD) [23] or MCI [24]. FACEHBI is a longitudinal observational study with the goal of investigating the pathophysiology of preclinical AD and the role of SCD as a risk marker for the future development of cognitive impairment [21]. For the present study, data from FACEHBI 5th follow-up visit (v5) were analyzed. BIOFACE is a longitudinal observational study focused on the analysis of novel biomarkers (including plasma-derived exosomes) in early-onset MCI patients [22]. For the present study, data from BIOFACE baseline visit (v0) were analyzed.

The cognitive status of all participants was assessed using the Neuropsychological Battery of Fundació ACE (NBACE) [25, 26]. NBACE is a brief, comprehensive and easy to administer test battery to detect cognitive impairment in the adulthood that covers the following cognitive domains: processing speed, orientation, attention, verbal learning and memory, language, visuo-perception, praxis, and executive functions. Participants classified as SCD presented cognitive complaints, a completely normal performance in all subtest of NBACE according to age and years of education, and preserved autonomy in daily life activities. Participants classified as MCI presented cognitive complaints, abnormal performance (low scores according to published cutoffs) in one or more NBACE subtests and preserved autonomy in daily life activities (no dementia).

For all participants, the following data were collected: demographics (age, sex, education), presence of cardiovascular risk factors (heart disease, respiratory disease, hypertension, dyslipidemia, smoking, diabetes mellitus, and stroke), Mini-Mental State Examination (MMSE) [27] scores, syndromic diagnosis (SCD or MCI), amyloid status (positivity defined as Florbetaben (FBB) Centiloid > 13.5 [28] for FACEHBI participants and cerebrospinal fluid (CSF) Aβ42/Aβ40 ratio < 0.063 [29] for BIOFACE participants), apolipoprotein E (*APOE*) ε4 status, OCT-A (macular VD in the superficial plexus in four quadrants), and brain MRI (CV and atrophy measures).

### Neuro-ophthalmological evaluation

Study participants underwent a complete neuro-ophthalmological evaluation that was performed by an optometrist and lasted about 20 min. The evaluation comprised: (1) review of past ophthalmological diseases, treatments, and surgeries, (2) monocular visual acuity assessment with the participants wearing their habitual correction for refractive error using a pinhole occluder and the Early Treatment of Diabetic Retinopathy Study (ETDRS) chart [30, 31], (3) intraocular pressure (IOP) measurement by Icare tonometry [32], and (4) swept source (SS) OCT scan. More details can be found elsewhere [33].

### Optical coherence tomography angiography

Participants were imaged with a DRI OCT Triton—SS OCT (Topcon Co. Tokyo, Japan). The OCT exam was completed in about 5–10 min, no pupil dilation was required and both eyes were scanned separately. Data were analyzed with the OCT Angiography Ratio Analysis (OCTARA) processing software. An automatic segmentation method was employed to obtain measures of the superficial vascular plexus, and the quantification of VD, expressed as the % of area covered by vessels. VD measures were obtained in a 6 × 6 mm area centered in the fovea. The central area (1 mm circle) was excluded from the analysis. The parafoveal area, defined by two concentric rings measuring 1- and 3-mm diameter, respectively, was subdivided into four quadrants: nasal, superior, temporal, and inferior. More details can be found in a recent publication [34]. Only VD measures from the right eye were used for the analysis, as in previous papers from our group [20, 33, 35].

### Brain magnetic resonance imaging acquisition and processing

All participants underwent a structural MRI within a 6-month window after the clinical assessment and OCT-A. Imaging data were analyzed using the Ace Alzheimer Center Barcelona Pipeline for Neuroimaging Analysis.

For FACEHBI participants, MRIs were acquired on a 1.5-T Siemens Magneton Aera (Erlangen, Germany) using a 32-channel head coil from Clínica Corachan (Barcelona). Anatomical T1-weighted images were acquired using a rapid acquisition gradient-echo three-dimensional (3D) magnetization-prepared rapid gradient-echo (MPRAGE) sequence with the following parameters: repetition time (TR) 2.200 ms, echo time (TE) 2.66 ms, inversion time (TI) 900 ms, slip angle 8°, field of view (FOV) 250 mm, slice thickness 1 mm, and isotropic voxel size 1 × 1 × 1 mm. Subjects also received axial T2-weighted, 3D isotropic fast fluid-attenuated inversion recovery (FLAIR), and axial T2*-weighted sequences to detect significant vascular pathology or microbleeds.

For BIOFACE participants, MRIs were acquired in a Siemens MAGNETOM VIDA 3 T scanner (Erlangen, Germany) using 32-channel head coil from Clinica Corachan (Barcelona). T1-weigthed images, for the morphological and the volumetric studies, were acquired using a gradient-echo 3D MPRAGE sequence with the following parameters: TR 2.200 ms, TE 2.23 ms, TI 968 ms, 1.2-mm slice thickness, FOV 270 mm, and voxel measurement 1.1 × 1.1 × 1.2 mm. In order to complete the acquisition a 3D isotropic FLAIR, an axial sequence T2-weighted, and an axial sequence T2*-weighted will be performed to detect vascular brain damage and microbleeds.

1.5-T and 3-T MRI images from the two cohorts (FACEHBI and BIOFACE) were analyzed together, as the scanner resolution (1.5 T vs 3 T) only affects image resolution (accuracy of the measurement) not the measurement per se. Additionally, all images were converted to the 1-mm^3^ voxel resolution, so the differences in measurements were even less noticeable.

MRI images were stored in a picture archiving communication system (PACS) system and submitted to an automated process of de-identification.

Two parameters were used to assess cerebrovascular damage: the Fazekas scale [36] and the volume of white matter hyperintensities (WMH).

The Fazekas scale is a visual assessment scale that divides the white matter into periventricular and deep white matter [36]. Each region is given a grade depending on the size and confluence of the lesions: (a) periventricular white matter (PVWM) (0 = absent, 1 = caps or pencil-thin lining, 2 = smooth halo, 3 = irregular periventricular signal extending into the deep white matter) and (b) deep white matter (DWM) (0 = absent, 1 = punctate foci, 2 = beginning confluence, 3 = large confluent areas). Image quantification using the Fazekas scale was performed by two expert radiologists who were blind to demographic characteristics and diagnostic status of participants. For the present analysis, as the Fazekas scale a high positive asymmetric distribution, scores were dichotomized into two categories: absence (Fazekas 0–1) and presence (Fazekas 2–3) of significant CV pathology.

**Fig. 1:**
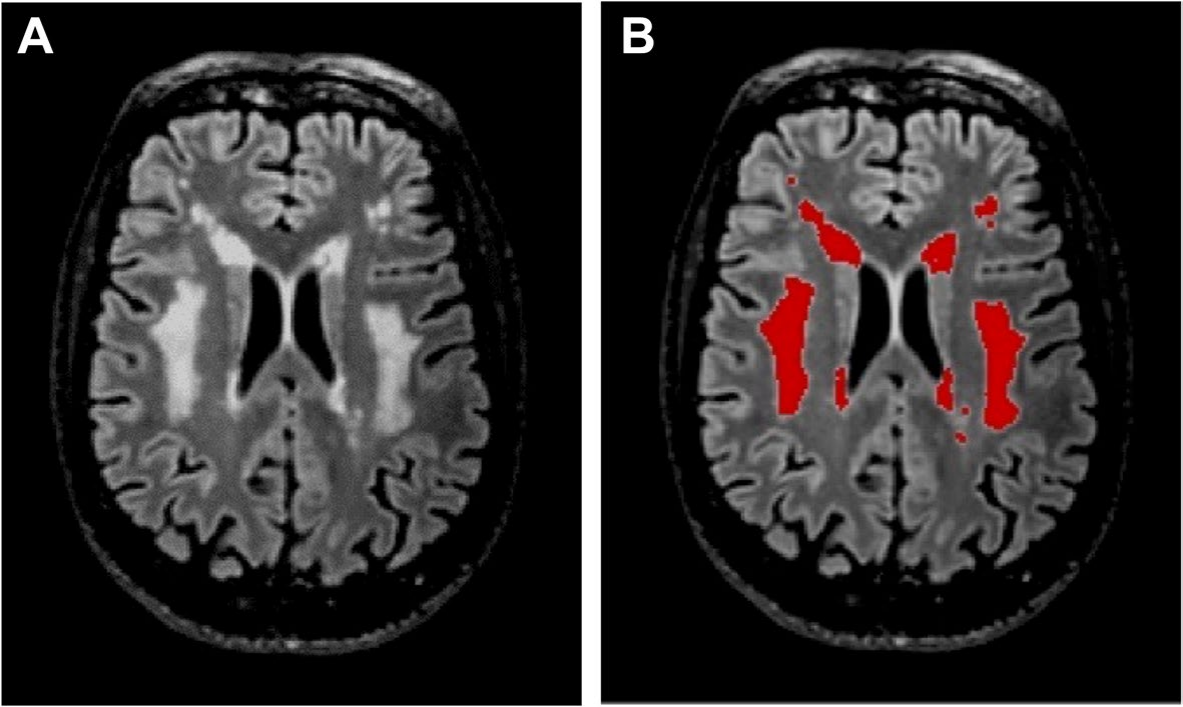
Representative MRI T2-weighted images from a 68-year-old male with SCD showing **A** substantial WMH damage (Fazekas scale score = 3) and **B** isolation and masking of the WMH burden (in red) using the PGS software. Abbreviations: MRI: magnetic resonance imaging; WMH: white matter hyperintensitiy; SCD: subjective cognitive decline

The volume of WMH was calculated as follows. First, T2-FLAIR was registered into T1w native space with Advanced Normalization Tools (ANTs) 3.0 software package [37]. Then with T1w and T2-FLAIR registered images, we used the U-Net with multi-scale highlighting foregrounds method described elsewhere [38] and the PGS software provided by the authors. The software isolates the WMH regions so the WMH volume could be straightforwardly obtained with FMRIB Software Library v6.0 tools [39] (Fig. 1). No partial volume correction was applied for the determination of WMH volume, since it should be equal to zero in a healthy brain regardless of the intracranial volume. Additionally, sex is the main contributor to the variation in intracranial volume and it was included as an adjusting factor in all the analysis.

Additionally, we calculated several structural brain parameters related to AD atrophy patterns: the hippocampal volume, the ventricle volume, and the Alzheimer’s disease cortical signature (ADCS) thickness. MRI T1w images underwent a Freesurfer 7.2 reconstruction [40–42]. Hippocampal volume was calculated as the sum of right and left hippocampus volume and adjusted by the estimated intracranial volume using the residual method [43]. Ventricle volume was calculated as the sum of the reconstructed ventricles and adjusted by the estimated intracranial volume using the residual method. Lastly, the ADCS was expressed by a meta-region of interest (ROI) composed of the following areas: entorhinal, inferior temporal, middle temporal, and fusiform. The average thickness of the ADCS ROI was calculated as the mean thickness across these regions weighted by their surface area [44–46].

### Statistical analyses

Data processing and analysis were carried out using R 4.1.2 [47]. All quantitative data were checked for normality, skewness, and range restriction. A log transformation was applied to those measures that did not follow a normal distribution (WMH burden and ventricle volume). Demographic and clinical variables (age, sex, education, APOE ε4 status, MMSE score, hypertension, diabetes mellitus, dyslipidemia, heart disease, respiratory disease, smoking, and stroke) were examined using frequency analysis (Student’s *t* and Pearson’s chi-square tests) to characterize their distribution between the two diagnostic groups (SCD vs MCI). In the following analyses, only those variables which prevalence was greater than 5% of the total cohort were included.

For the final models, we included cardiovascular variables (hypertension, diabetes mellitus, dyslipidemia, heart disease, respiratory disease, and smoking) as adjusting factors. In order to determine which demographic and clinical variables should be additionally included as adjusting factors in the models, four multiple linear regression analyses were carried out to study their distribution among the four regional macular VD measures, separately. We examined the following variables: age, sex, education, *APOE* ε4 status, amyloid (A) status, and syndromic diagnosis (SCD vs MCI), using cardiovascular variables (hypertension, dyslipidemia, diabetes mellitus, heart disease, respiratory disease, and smoking) as adjusting factors. For all the analyses, alpha level was set at 0.05.

Five linear regression analyses were performed to determine the capacity of each regional macular VD measure to discriminate brain vascular and structural changes. Those demographic and clinical variables that showed a significant impact in the previous multiple linear regression analyses, along with cardiovascular variables (heart disease, respiratory disease, hypertension, dyslipidemia, smoking, and diabetes mellitus), were included as adjusting factors in the analyses. All the regression analyses described below were performed as follows: first, we ran the analyses without the adjusting factors, and then introduced those to assess their effect on the discrimination task.

First, a logistic regression analysis of the association of regional VD measures between the Fazekas categories was performed. We reported the odds ratio and its 95% confidence interval, the *z*-scores, and significance. Then, four different multiple linear regression analyses of the association of each regional macular VD measure with the WMH volume, hippocampal volume, ventricle volume, and the ADCS thickness as outcomes, separately, were performed. For these analyses, we reported regression coefficients, *t*-value, significance, and beta. Alpha level was set at 0.0125 after Bonferroni correction.

Lastly, the former analyses were repeated to assess the interaction between the A status and the four regional VD measures to discriminate brain vascular (Fazekas categories, WMH volume) and structural (hippocampal volume, ventricle volume and ADCS thickness) features, separately. Alpha level was set at 0.0125 after Bonferroni correction.

## Results

### Demographic and clinical characteristics of the cohort

**Fig. 2:**
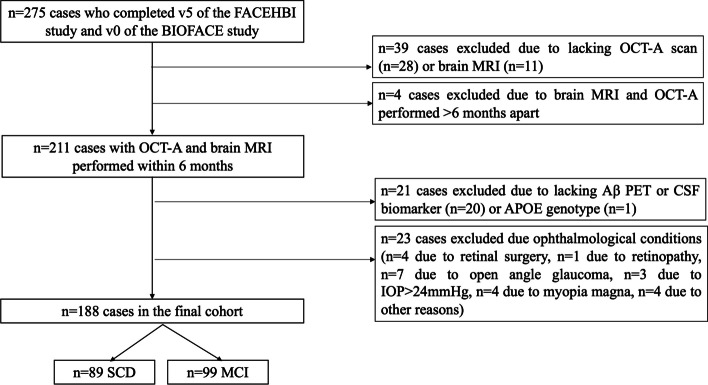
Study flowchart. Abbreviations: APOE: apolipoprotein E; CSF: cerebrospinal fluid; IOP: intraocular pressure; MCI: mild cognitive impairment; MRI: magnetic brain imaging; OCT-A: Optical Coherence Tomography – angiography; PET: positron emission tomography; SCD: subjective cognitive decline

Data from 275 participants who completed v5 of the FACEHBI study and v0 of the BIOFACE study were initially reviewed (Fig. 2). Several exclusion criteria were applied: lack of OCT-A measures (*n* = 28), lack of brain MRI (*n* = 11), time between OCT-A and brain MRI > 6 months (*n* = 4), lack of Aβ positron emission tomography (PET) or CSF biomarkers (*n* = 21), lack of *APOE* genotype (*n* = 1), and finally, ophthalmological conditions that could interfere with the OCT-A measurements (*n* = 23: *n* = 4 due to retinal surgery, *n* = 1 due to retinopathy, *n* = 7 due to open-angle glaucoma, *n* = 3 due to IOP > 24 mmHg, *n* = 4 due to other reasons)).

The final sample consisted of 188 individuals (89 with SCD and 99 with MCI) who completed FACEHBI v5 or BIOFACE v0 and had OCT-A, brain MRI, amyloid status, and *APOE* genotype information available.

Demographic characteristics and past medical history of participants are displayed in Table 1. Compared to SCD individuals, those in the MCI group showed younger age (64.35 ± 6.82 vs 68.47 ± 7.27, *p* < 0.001), fewer years of education (12.41 ± 5.11 vs 15.71 ± 4.24, *p* < 0.001), lower MMSE scores (27.91 ± 1.64 vs 29.07 ± 1.15, *p* < 0.001), lower rates of respiratory disease (6.06% vs 16.85%, *p* = 0.035), and higher rates of smoking (49.49% vs 22.47%, *p* < 0.001).

**Table 1:** Demographic and clinical characteristics of the study cohort

Variables	Whole cohort (*n* = 188)	SCD (*n* = 89)	MCI (*n* = 99)	Significance
Age (year)	66.30 ± 7.32	68.47 ± 7.27	64.35 ± 6.82	< 0.001* ^a^
Sex (females)	119 (63.30%)	59 (66.29%)	60 (60.61%)	0.512 ^b^
Education (year)	13.97 ± 4.98	15.71 ± 4.24	12.41 ± 5.11	< 0.001* ^a^
APOE ε4 status	58 (30.85%)	23 (25.84%)	35 (35.35%)	0.211 ^b^
MMSE score	28.46 ± 1.54	29.07 ± 1.15	27.91 ± 1.64	< 0.001* ^a^
Hypertension	63 (33.51%)	33 (37.08%)	30 (30.30%)	0.408 ^b^
Diabetes mellitus	11 (5.85%)	4 (4.94%)	7 (7.07%)	0.660 ^b^
Dyslipidemia	76 (40.43%)	39 (43.82%)	37 (37.37%)	0.453 ^b^
Heart disease	19 (10.11%)	10 (11.24%)	9 (9.09%)	0.807 ^b^
Respiratory disease	21 (11.17%)	15 (16.85%)	6 (6.06%)	0.035* ^b^
Stroke	3 (1.60%)	0 (0.00%)	3 (3.03%)	0.258 ^b^
Smoking	69 (36.70%)	20 (22.47%)	49 (49.49%)	< 0.001* ^b^

Data are shown as mean ± standard deviation for quantitative variables and *n* (%) for qualitative variables

*Abbreviations*: *APOE* Apolipoprotein E, *MCI* Mild cognitive impairment, *MMSE* Mini-Mental State Examination, *SCD* Subjective cognitive decline

^*^Significance was set up at *p* < 0.05

^a^Student’s *t* test; ^b^Pearson’s chi-square test

Regarding biomarker data (see Table 2), MCI and SCD participants showed similar rates of amyloidosis (25.25% vs 31.46%, *p* = 0.434) and CV damage (Fazekas category 2–3: 7.07% vs 14.62%, *p* = 0.151; WMH volume (3020.31 ± 4289.90 vs 3408.14 ± 4752.04, *p* = 0.559)). MCI participants showed significantly higher regional macular VD measures compared to the SCD group in the nasal, temporal, and inferior quadrants (*p* = 0.029, *p* < 0.001, and *p* = 0.024, respectively).

**Table 2:** Biomarker data of the study cohort

Variables	Whole cohort (*n* = 188)	SCD (*n* = 89)	MCI (*n* = 99)	Significance
A + status	53 (28.19%)	28 (31.46%)	25 (25.25%)	0.434 ^b^
Brain MRI				
Fazekas category 2–3	20 (10.64%)	13 (14.61%)	7 (7.07%)	0.151 ^b^
WMH volume (mm^3^)	3203.91 ± 4506.54	3408.14 ± 4752.04	3020.31 ± 4289.90	0.559 ^a^
OCT-A				
VD nasal	47.66 ± 3.81	47.03 ± 3.16	48.23 ± 4.24	0.028* ^a^
VD superior	48.67 ± 4.74	48.11 ± 4.18	49.17 ± 5.16	0.124 ^a^
VD temporal	45.83 ± 3.42	44.97 ± 2.94	46.59 ± 3.65	0.001* ^a^
VD inferior	47.81 ± 5.44	46.89 ± 4.40	48.65 ± 6.14	0.024* ^a^

Data are shown as mean ± standard deviation for quantitative variables and *n* (%) for qualitative variables

A + status was defined as FBB-PET Centiloid > 13.5 in the FACEHBI study and CSF Aβ42/Aβ40 ratio < 0.063 in the BIOFACE study

*Abbreviations*: *A* Amyloid, *MCI* Mild cognitive impairment, *MRI* Magnetic resonance imaging, *OCT-A* Optical coherence tomography – angiography, *SCD* Subjective cognitive decline, *VD* Vessel density, *WMH* White matter hyperintensity

^*^Significance was set up at *p* < 0.05

^a^Student’s *t* test; ^b^Pearson’s chi-square test

### Multiple linear regression analyses of clinical, demographic, and biomarker variables with regional VD measures

The multiple linear regression analysis exploring the association of age, sex, education, *APOE* ε4 status, A status, and syndromic diagnosis (SCD vs MCI) with each regional macular VD measure showed that age had a significant effect on macular VD in the nasal, temporal, and inferior quadrants (all, *p* < 0.027), sex had a significant effect on macular VD in the temporal quadrant (*p* = 0.033), and syndromic diagnosis had a significant effect on macular VD in the temporal and superior quadrants (*p* < 0.043), so those were included as adjusting factors in all following analyses (Additional file 1).

### Logistic regression analysis of the association of macular VD and Fazekas categories

**Fig. 3:**
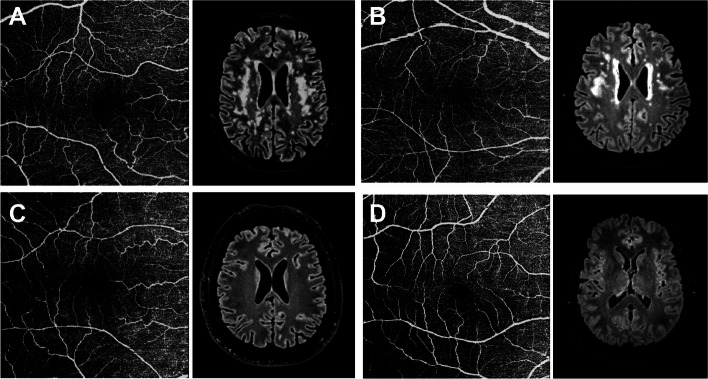
Representative OCT-A and brain MRI images from the study participants. **A** 68-year-old female with SCD and high CV damage (Fazekas scale score = 2, WMH volume = 30,943 mm^3^). **B** 61-year-old female with MCI and high CV damage (Fazekas scale score = 3, WMH volume = 27,000 mm^3^). **C** 61-year-old female with SCD and low CV damage (Fazekas scale score = 0, WMH volume = 290 mm^*3*^). **D** 60-year-old female with MCI and low CV damage (Fazekas scale score = 0, WMH volume = 710 mm^3^). Abbreviations: CV: cerebrovascular; MCI: mild cognitive impairment; MRI: magnetic ressonance imaging; OCT-A: Optical Coherence Tomography – angiography; SCD: subjective cognitive decline; WMH: white matter hyperintensity

Representative images of OCT-A and brain MRIs from study participants are depicted in Fig. 3.

The association of regional macular VD measures with the Fazekas categories is depicted in Tables 3 and 4, with and without including age, sex, syndromic diagnosis, and cardiovascular variables as adjusting factors.

**Table 3:** Logistic regression analysis of the association of macular VD and Fazekas categories including adjusting factors

Variables	OR^1^	95% CI^1^	z^1^	Significance^1^
Age	1.14	1.04–1.26	2.75	0.006*
Sex	2.32	0.69–9.27	1.29	0.196
Syndromic diagnosis	0.41	0.10–1.51	1.29	0.197
Hypertension	6.35	1.94–23.61	2.94	0.003*
Diabetes mellitus	1.34	0.17–7.37	0.32	0.751
Dyslipidemia	0.37	0.10–1.22	1.56	0.118
Heart disease	0.77	0.08–4.61	0.26	0.794
Respiratory disease	4.29	0.82–22.30	1.77	0.077
Smoking	7.01	1.89–30.93	2.78	0.005*
VD nasal	1.03	0.84–1.27	0.30	0.762
VD temporal	1.18	0.93–1.54	1.33	0.182
VD superior	0.91	0.80–1.02	1.60	0.111
VD inferior	1.04	0.93–1.15	0.67	0.503

Including age, sex, syndromic diagnosis, hypertension, diabetes mellitus, dyslipidemia, heart disease, respiratory disease, and smoking as adjusting factors

*Abbreviations*: *CI* Confidence interval, *OR* Odds ratio, *VD* Vessel density

^*^Significance was set up at *p* < 0.0125

**Table 4:** Logistic regression analysis of the association of macular VD and Fazekas categories not including adjusting factors

Variables	OR	95% CI	*z*	Significance
VD nasal	1.03	0.89–1.21	0.39	0.694
VD temporal	0.96	0.80–1.17	0.36	0.716
VD superior	0.93	0.84–1.03	1.49	0.137
VD inferior	1.03	0.95–1.12	0.79	0.428

*Abbreviations*: *CI* Confidence interval, *OR* Odds ratio, *VD* Vessel density

^*^Significance was set up at *p* < 0.0125

**Fig. 4:**
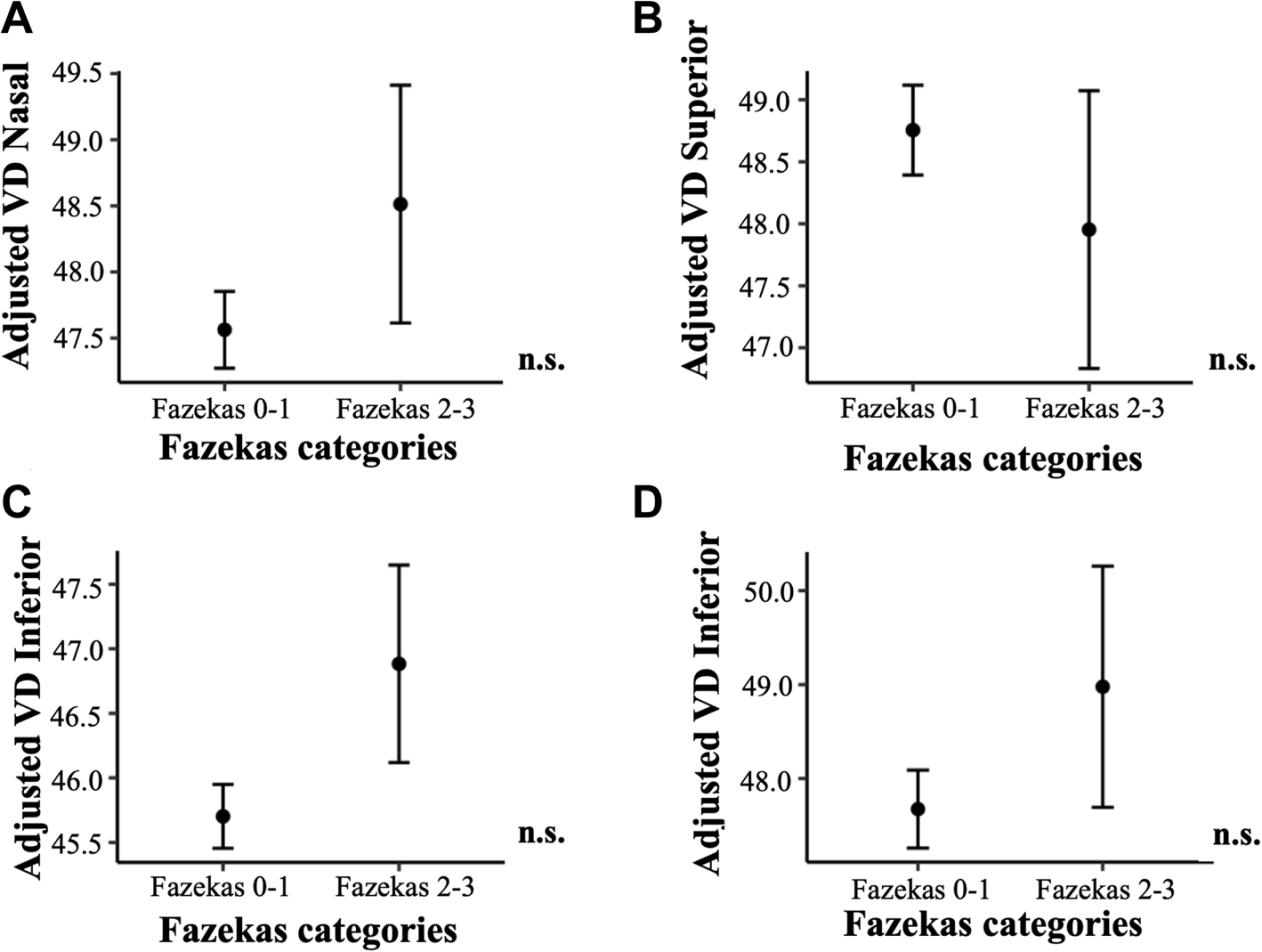
Adjusted macular VD measurements by Fazekas categories. Macular VD differences between Fazekas categories in **A** nasal, **B** superior, **C** temporal, and **D** inferior quadrants. Macular VD measures are adjusted by age, sex, syndromic diagnosis, hypertension, diabetes mellitus, dyslipidemia, heart disease, respiratory disease, and smoking. Abbreviations: n.s.: non-significant; VD: vessel density

Regression models revealed that regional macular VD measures were not able to discriminate the Fazekas categories (all *p* > 0.111, Fig. 4). Age (*p* = 0.006), hypertension (*p* = 0.003), and smoking (*p* = 0.005) showed a significant association with the Fazekas categories.

### Multiple linear regression analysis of the association of macular VD with WMH volume

**Fig. 5:**
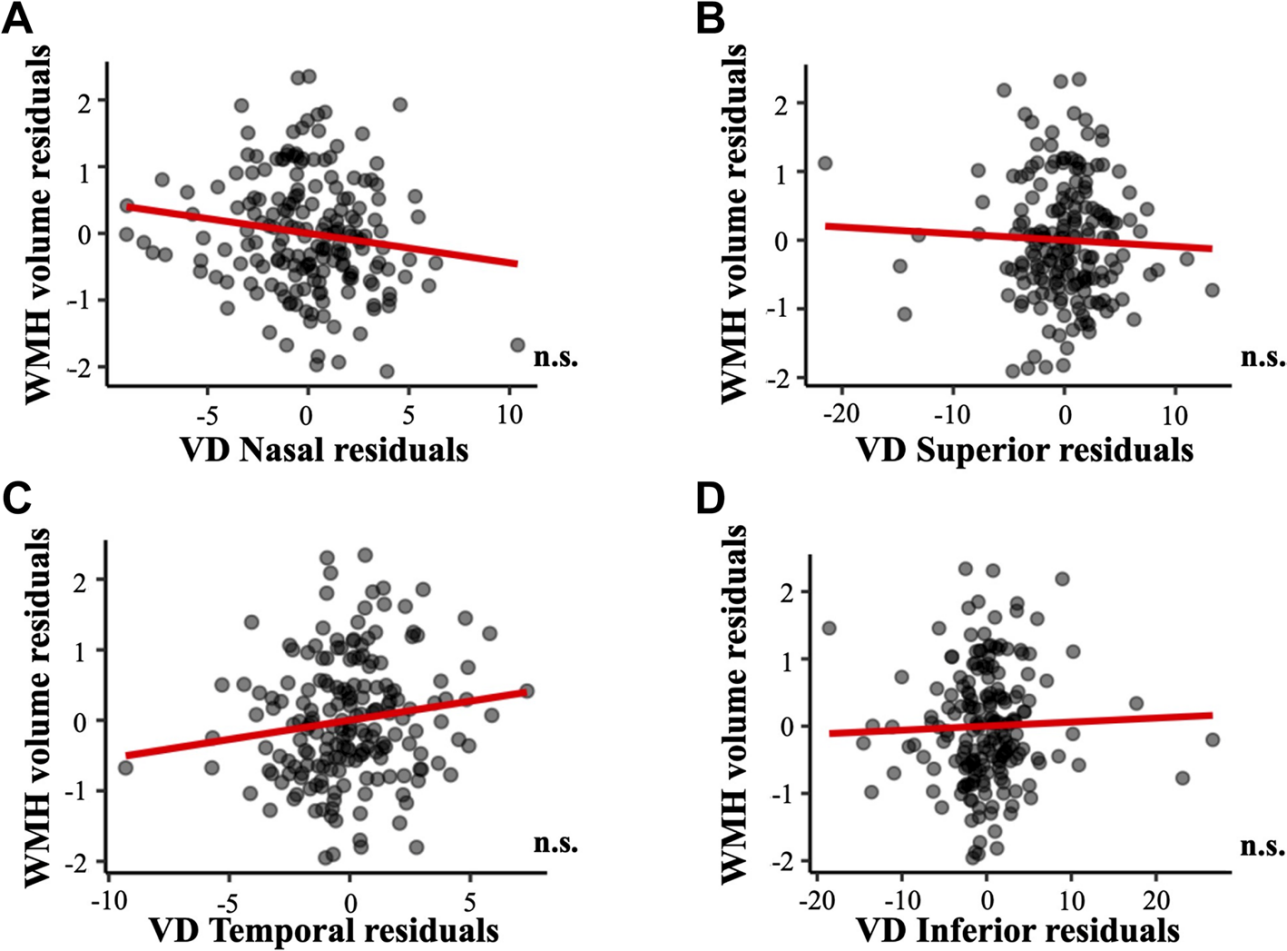
Association of macular VD and WMH volume. Association of macular VD in **A** nasal, **B** superior, **C** temporal, and **D** inferior quadrants with WHM volume. Macular VD measures are adjusted by age, sex, syndromic diagnosis, hypertension, diabetes mellitus, dyslipidemia, heart disease, respiratory disease, and smoking. Abbreviations: n.s.: non-significant; VD: vessel density; WMH: white matter hyperintensity

The association of regional macular VD measures with WMH volume, with and without including age, sex, syndromic diagnosis, and all cardiovascular variables as adjusting factors, are shown in Tables 5 and 6. Regression models revealed that regional macular VD was not associated to WMH volume (all, *p* > 0.051, Fig. 5). Age showed a positive association with the WMH volume (*p* < 0.001).

**Table 5:** Multiple linear regression analysis of the association of macular VD and WMH volume including adjusting factors

Variables	Coefficient	*t*	Significance	Beta
Age	0.05	4.83	< 0.001*	0.37
Sex	0.06	0.41	0.685	0.03
Syndromic diagnosis	0.20	1.40	0.163	0.10
Hypertension	0.34	2.28	0.024	0.16
Diabetes mellitus	0.60	2.09	0.038	0.14
Dyslipidemia	− 0.06	0.44	0.660	− 0.03
Heart disease	0.09	0.38	0.702	0.03
Respiratory disease	0.38	1.75	0.082	0.12
Smoking	− 0.03	0.20	0.845	− 0.01
VD nasal	− 0.04	1.97	0.051	− 0.17
VD temporal	0.05	1.93	0.056	0.19
VD superior	− 0.01	0.58	0.563	− 0.05
VD inferior	0.01	0.47	0.640	0.03

Including age, sex, syndromic diagnosis, hypertension, diabetes mellitus, dyslipidemia, heart disease, respiratory disease, and smoking as adjusting factors

A log transformation was applied to WMH volume measures

*Abbreviations*: *VD* Vessel density, *WMH* White matter hyperintensities

^*^Significance was set up at *p* < 0.0125

**Table 6:** Multiple linear regression analysis of the association of macular VD and WMH volume not including adjusting factors

Variables	Coefficient	t	Significance	Beta
VD nasal	− 0.04	1.83	0.068	− 0.17
VD temporal	0.02	0.53	0.595	0.05
VD superior	− 0.00	0.19	0.852	− 0.02
VD inferior	− 0.00	0.23	0.816	− 0.02

A log transformation was applied to WMH volume measures

*Abbreviations*: *VD* Vessel density, *WMH* White matter hyperintensities

^*^Significance was set up at *p* < 0.0125

### Multiple linear regression analysis of the association of macular VD with brain atrophy measures

**Fig. 6:**
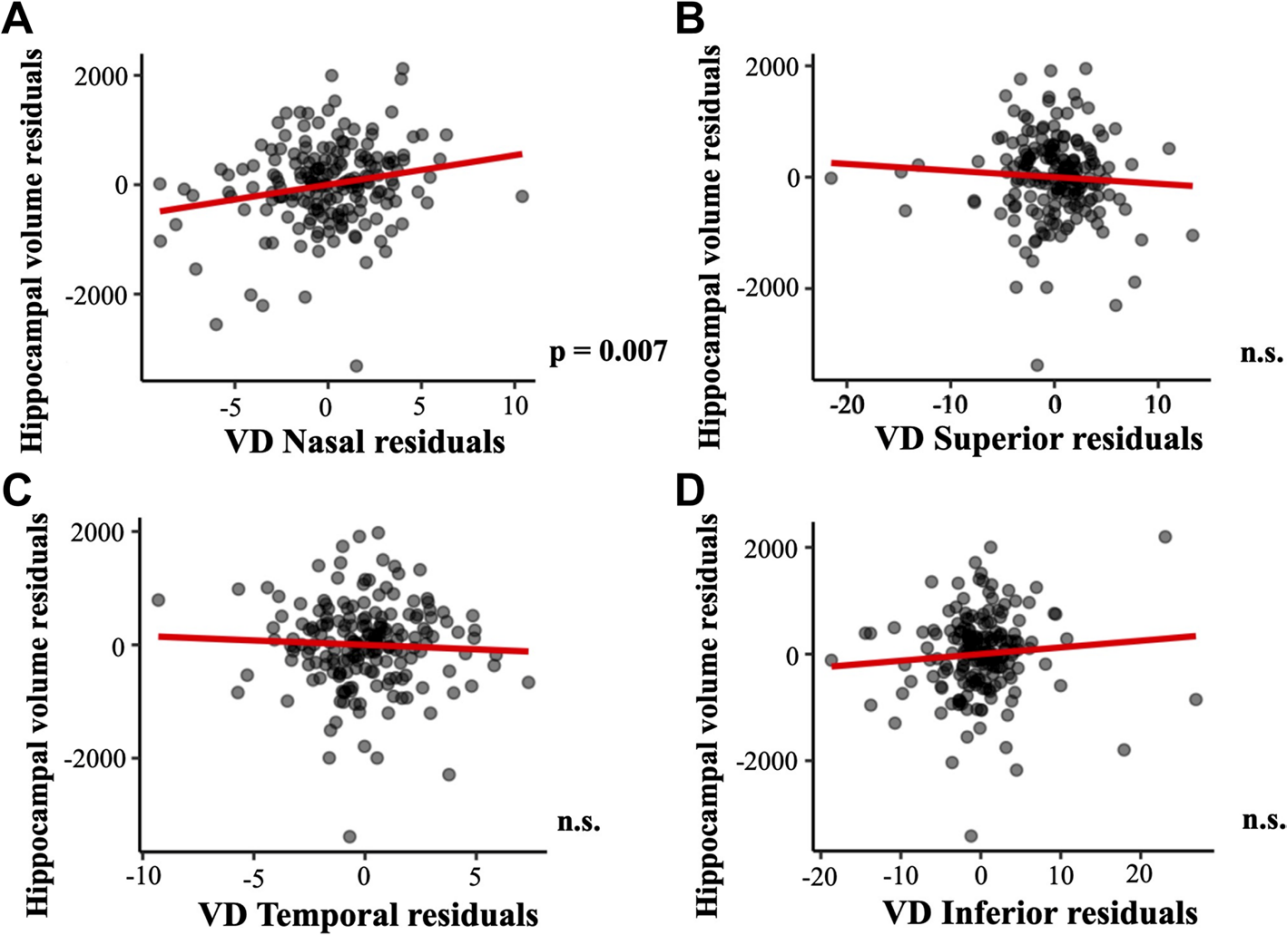
Association of macular VD and hippocampal volume. Association of macular VD in **A** nasal, **B** superior, **C** temporal, and **D** inferior quadrants with hippocampal volume. Macular VD measures are adjusted by age, sex, syndromic diagnosis, hypertension, diabetes mellitus, dyslipidemia, heart disease, respiratory disease, and smoking. Abbreviations: n.s.: non-significant; VD: vessel density; WMH: white matter hyperintensity

**Fig. 7:**
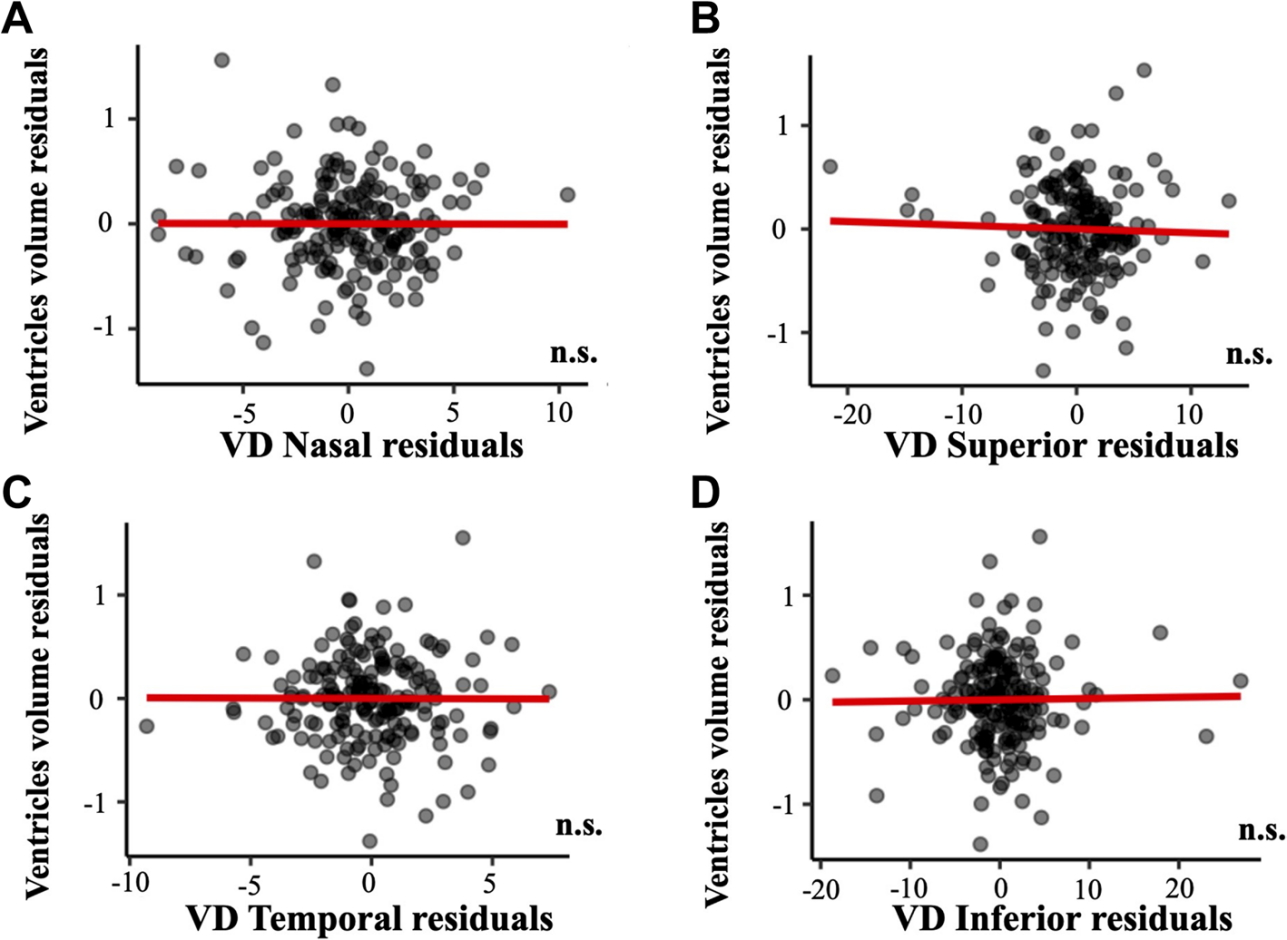
Association of macular VD and ventricle volume. Association of macular VD in **A** nasal, **B** superior, **C** temporal, and **D** inferior quadrants with ventricle volume. Macular VD measures are adjusted by age, sex, syndromic diagnosis, hypertension, diabetes mellitus, dyslipidemia, heart disease, respiratory disease, and smoking. Abbreviations: n.s.: non-significant; VD: vessel density

**Fig. 8:**
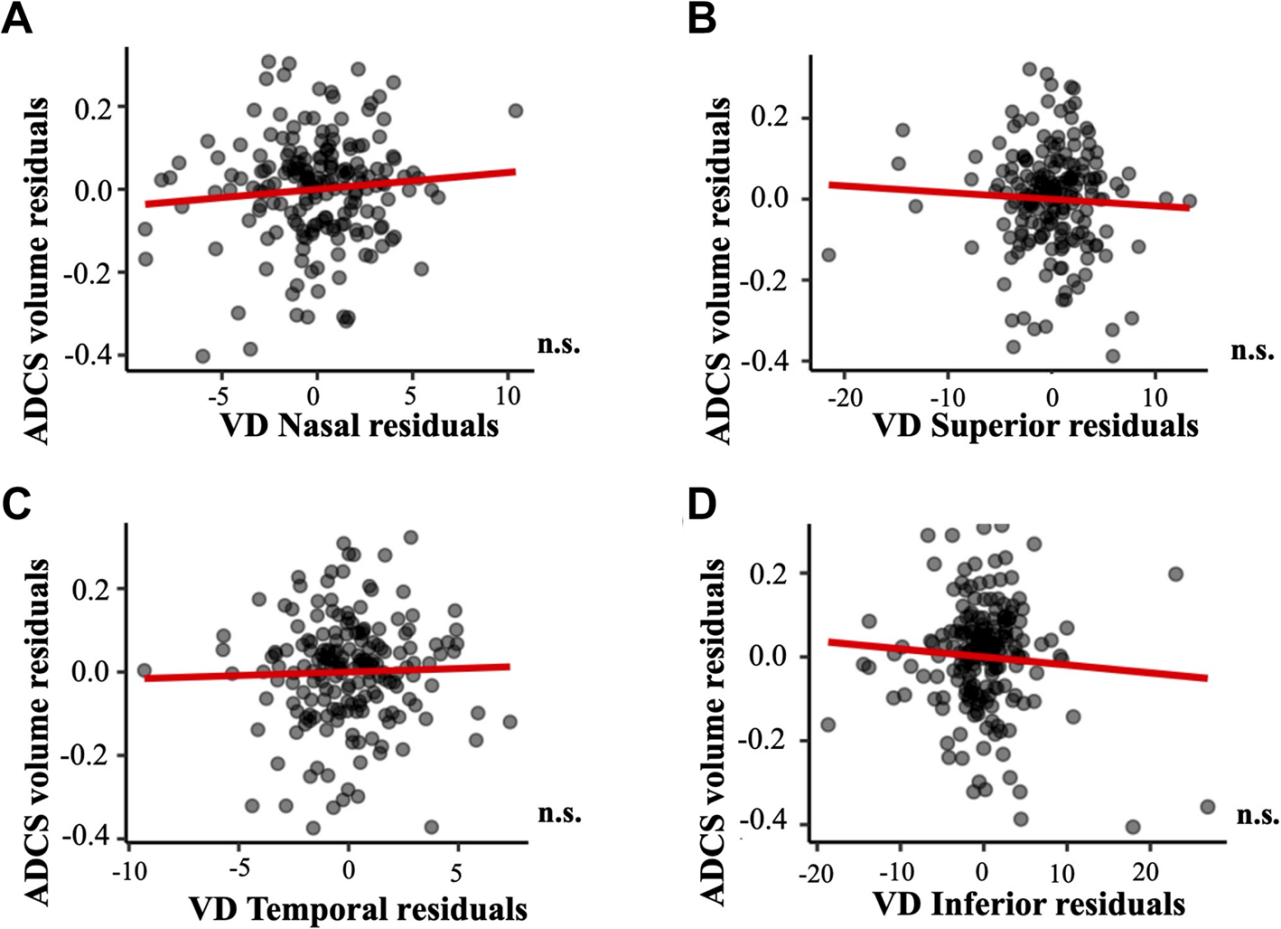
Association of macular VD and ADCS thickness. Association of macular VD in **A** nasal, **B** superior, **C** temporal, and **D** inferior quadrants with ACDS thickness. Macular VD measures are adjusted by age, sex, syndromic diagnosis, hypertension, diabetes mellitus, dyslipidemia, heart disease, respiratory disease, and smoking. Abbreviations: ADCS: Alzheimer’s disease cortical signature; non-significant; VD: vessel density

The associations of regional macular VD with brain atrophy measures, with and without including age, sex, syndromic diagnosis, and cardiovascular variables as adjusting factors, are depicted in Additional files 2, 3, 4, 5, 6 and 7. Regression models showed that VD in the nasal quadrant was significantly associated to hippocampal volume (*p* = 0.007, Fig. 6). On the other hand, VD measures were not associated to other measures of brain atrophy such as ventricle volume (all, *p* > 0.657, Fig. 7) or ADCS thickness (all, *p* > 0.235, Fig. 8). Age showed a negative association with hippocampal volume (*p* < 0.001, Additional file 2) and a positive association with ventricle volume (*p* < 0.001, Additional file 4). Diabetes showed a positive association with ventricle volume (*p* = 0.001, Additional file 4). Lastly, syndromic diagnosis showed a negative association with ADCS thickness (*p* < 0.001, Additional file 6).

### Interaction of the A status and macular VD in discriminating CV damage and brain atrophy

Regression models showed that the interaction of the A status with macular VD had no effect in differentiating Fazekas categories (all *p* > 0.079, Additional file 8), WMH volume (all *p* > 0.374, Additional file 9), hippocampal volume (all *p* > 0.328, Additional file 10), ventricle volume (all *p* > 0.410, Additional file 11), or ADCS thickness (all *p* > 0.065, Additional file 12)*.* Thus, the association of macular VD with measures of CV damage and brain atrophy was not significantly influenced by the A status.

## Discussion

We investigated the relationship of macular VD in the superficial plexus quantified by OCT-A with measures of CV pathology and brain atrophy in 188 non-demented participants from two research cohorts at Ace Alzheimer Center Barcelona. Our data suggested that macular VD was not a proxy of CV burden but was significantly associated with hippocampal atrophy independently of amyloid status.

In the dementia field, the early identification of CV pathology is crucial, as asymptomatic brain vascular changes in middle-aged adults are associated with a higher risk of future cognitive decline and disability [48]. Preliminary data suggested that OCT-A had the potential to be used as an alternative, direct, and non-invasive method to assess the health of brain vasculature in early stages of CV damage instead of conventional imaging techniques such as brain MRI, which has a limited diagnostic accuracy and does not allow the in vivo visualization of brain microvasculature. Two publications from large cohorts supported this idea, showing significant associations between vascular retinal changes and CV pathology. First, changes in retinal vascular calibers (narrower arterioles and wider venules) from fundus photographs showed an association with poor white matter microstructure on diffusor tension MRI [49]. Second, subclinical cerebral infarcts were associated with retinal microvascular abnormalities (arteriovenous nicking, focal arteriolar narrowing, blot hemorrhages, soft exudates, and microaneurysms) in individuals with hypertension [50]. Additionally, several smaller studies also highlighted a similar association between retinal and brain vascular pathology [51–58]. Our current results, though, do not support the correlation of retinal *vs* brain vascular changes, as we did not detect a significant association of macular VD with the Fazekas scale scores and WMH volume assessed by brain MRI. We believe that the discrepancy between our data and results from previous publications might be due to several factors. First of all, one of the main methodological differences among published data are the measures used to assess retinal and brain vascular damage, which differ widely among works (for retinal measures: retinal vascular calibers, arteriovenous nicking, arteriolar narrowing, arterial tortuosity, hemorrhages, exudates, microaneurysms, wall-to-lumen arterial ratio, skeleton density of capillaries, VD, arterial fractal dimension; for brain measures: diffusor tensor for WM microstructure assessment, presence of cerebral infarcts, volume of WMH, markers of vascular integrity, CV reactivity in WM …). In particular, in our study, we used a quantitative measure of retinal vascular pathology (macular VD in the superficial vascular plexus in four different quadrants) and both semi-quantitative and quantitative data to measure CV damage (the Fazekas scale and WMH volume by Freesurfer, respectively), which were not directly comparable with any other publications. Additionally, the type of population (with and without cognitive impairment, with or without CV risk factors, with or without stroke) and number of participants included also varied among studies and could be influencing the outcomes. The types of retinal devices (OCT vs fundus photography) and brain MRI used for the vascular quantifications could play a part in the discrepant results among publications. Lastly, the methods used in our publication might not be sensitive enough to detect very small effects in the association of retinal and brain vascular measures.

It is well known that CV damage and neurodegeneration develop in parallel, thus we aimed to additionally investigate the relationship of retinal vascular changes with measures of brain atrophy. Our study detected that retinal vascular loss in the nasal quadrant (lower VD) was significantly associated with hippocampal atrophy, while the other measures of brain atrophy investigated (ventricle volume and ADCS thickness) were not related to retinal vascular changes. Although multiple works have demonstrated a significant association between retinal structural changes (retinal nerve fiber layer (RNFL) and macular thinning mostly) and brain atrophy [59–64], few publications have focused on the specific relationship of retinal vasculature with brain structural changes. Similar to our results, Hu et al. showed a positive association between retinal VD and gray matter volumes of the hippocampal subfields in 25 cognitively impaired patients (17 MCI and 8 AD dementia) [65].

Finally, our data showed that the association of macular VD with measures of CV damage and brain atrophy was not significantly influenced by the brain amyloid status of the participants, assessed by either CSF of PET. This particular issue had not been clearly addressed in previous publications correlating retinal and brain vascular damage, which either did not report the amyloid status of participants [48–54] or analyzed altogether data of participants with presence and absence of brain amyloidosis [57]. Our result also fits with a previous publication from our group using data from the NORFACE cohort, showing that macular VD did not significantly differ between Alzheimer and Normal AT(N) categories assessed by CSF biomarkers, and that macular VD was not correlated with CSF Aβ1-42 [66].

We acknowledge that our study has limitations. First of all, our cohort had a relatively low burden of CV pathology (only 10.64% of the sample belonged to the Fazekas 2–3 group). Second, our data was obtained from two research studies, not necessarily reflecting retinal and brain vascular changes of real-world patients with cognitive decline. Third, our results were cross-sectional, not being able to show changes over time in macular VD or brain MRI measures. Forth, the VD measures employed were limited to the macular region and the superficial vascular plexus, lacking information about FAZ and VD in the deep vascular plexus. Lastly, we lacked information about CV-related drugs taken by the study participants and the quality of the OCT-A images.

We also consider that our study has several strengths compared to previous works. Our cohort consisted of a relatively large and single-site sample of non-demented research participants who underwent similar testing and biomarkers. We limited our analysis to data from the right eye. Our participants’ age range was quite large (51–91), allowing us to potentially detecting macular VD changes in early and late ages. Notably, we used several cardiovascular risk factors as adjusting factors in all the analysis. Lastly, the neurologist and optometrist were blinded of each other’s diagnosis.

In summary, our study does not support that macular VD in the superficial plexus assessed by OCT-A is a proxy of CV damage in a research cohort of non-demented individuals with SCD and MCI.

### Supplementary Information

**Additional file 1.** Multiple linear regression analyses of the association of clinical, demographic and biomarker variables with macular VD. Including hypertension, diabetes mellitus, dyslipidemia, heart disease, respiratory disease and smoking as adjusting factors. Significance was set up at *p* < 0.05. Abbreviations: A: amyloid; APOE: apolipoprotein E; CI: confidence interval; VD: vessel density.

**Additional file 2.** Multiple linear regression analysis of the association of regional macular VD with hippocampal volume with adjusting factors. Including age, sex, syndromic diagnosis, hypertension, diabetes mellitus, dyslipidemia, heart disease, respiratory disease and smoking as adjusting factors. Significance was set up at *p* < 0.0125. Abbreviation: VD: vessel density.

**Additional file 3.** Multiple linear regression analysis of the association of regional macular VD with hippocampal volume without adjusting factors. Significance was set up at *p* < 0.0125. Abbreviation: VD: vessel density.

**Additional file 4.** Multiple linear regression analysis of the association of regional macular VD with ventricle volume with adjusting factors. Including age, sex, syndromic diagnosis, hypertension, diabetes mellitus, dyslipidemia, heart disease, respiratory disease and smoking as adjusting factors. Significance was set up at *p* < 0.0125. Abbreviation: VD = vessel density. Note: A log transformation was applied to the ventricles volume measures.

**Additional file 5:** Multiple linear regression analysis of the association of regional macular VD with ventricles volume without adjusting factors. Significance was set up at *p* < 0.0125. Abbreviation: VD = vessel density. Note: A log transformation was applied to the ventricles volume measures.

**Additional file 6.** Multiple linear regression analysis of the association of regional macular VD with ADCS thickness with adjusting factors. Including age, sex, syndromic diagnosis, hypertension, diabetes mellitus, dyslipidemia, heart disease, respiratory disease and smoking as adjusting factors. Significance was set up at *p* < 0.0125. Abbreviations: ADCS: Alzheimer´s disease cortical signature; VD = vessel density.

**Additional file 7.** Multiple linear regression analysis of the association of regional macular VD with ADCS thickness without adjusting factors. Significance was set up at *p* < 0.0125. Abbreviations: ADCS: Alzheimer´s disease cortical signature; VD = vessel density.

**Additional file 8.** Logistic regression analysis of the interaction of the A status and macular VD in discriminating Fazekas categories. Including age, sex, syndromic diagnosis, hypertension, diabetes mellitus, dyslipidemia, heart disease, respiratory disease and smoking as adjusting factors. Significance was set up at *p* < 0.0125. Abbreviations: CI: confidence interval; OR: odds ratio; VD: vessel density.

**Additional file 9.** Multivariate regression analysis of the interaction of the A status and macular VD in discriminating WMH volume. Including age, sex, syndromic diagnosis, hypertension, diabetes mellitus, dyslipidemia, heart disease, respiratory disease and smoking as adjusting factors. Significance was set up at *p* < 0.0125. Abbreviations: A: amyloid; V: vessel density.

**Additional file 10.** Multivariate regression analysis of the interaction of the A status and macular VD in discriminating hippocampal volume. Including age, sex, syndromic diagnosis, hypertension, diabetes mellitus, dyslipidemia, heart disease, respiratory disease and smoking as adjusting factors. Significance was set up at *p* < 0.0125. Abbreviations: A: amyloid; VD: vessel density.

**Additional file 11.** Multivariate regression analysis of the interaction of the A status and macular VD in discriminating ventricles volume. Including age, sex, syndromic diagnosis, hypertension, diabetes mellitus, dyslipidemia, heart disease, respiratory disease and smoking as adjusting factors. Significance was set up at *p* < 0.0125. Abbreviations: A: amyloid; VD: vessel density. Note: A log transformation was applied to the ventricles volume measures.

**Additional file 12.** Multivariate regression analysis of the interaction of the A status and macular VD in discriminating ACDS thickness. Including age, sex, syndromic diagnosis, hypertension, diabetes mellitus, dyslipidemia, heart disease, respiratory disease and smoking as adjusting factors. Significance was set up at *p* < 0.0125. Abbreviations: A: amyloid; ADCS: Alzheimer´s disease cortical signature; VD: vessel density.

## Data Availability

Not Applicable.

## Abbreviations

3D: Three-dimensional
A: Amyloid
AD: Alzheimer disease
ADCS: Alzheimer disease cortical signature
ANT: Advanced normalization tools
APOE: Apolipoprotein E
CSF: Cerebrospinal fluid
CSVD: Cerebral small-vessel disease
CV: Cerebrovascular
DWM: Deep white matter
ETDRS: Early Treatment of Diabetic Retinopathy Study
FACEHBI: Fundació ACE Healthy Brain Initiative
FAZ: Foveal avascular zone
FBB: Florbetaben
FDA: Food and Drug Administration
FLAIR: Fluid-attenuated inversion recovery
FOV: Field of view
IOP: Intraocular pressure
MCI: Mild cognitive impairment
MMSE: Mini-Mental State Examination
MPRAGE: Magnetization-prepared rapid gradient-echo
MRI: Magnetic resonance imaging
NBACE: Neuropsychological Battery of Fundació ACE
NORFACE: Neuro-ophthalmology Research at Fundació ACE
OCT-A: Optical coherence tomography angiography
OCTARA: OCT Angiography Ratio Analysis
PACS: Picture archiving communication system
PET: Positron emission tomography
PVWM: Periventricular white matter
RNFL: Retinal nerve fiber layer
ROI: Region of interest
SCD: Subjective cognitive decline
SS: Swept source
TE: Echo time
TI: Inversion time
TR: Repetition time
V0: Baseline visit
V5: 5th FACEHBI follow-up visit
WMH: White matter hyperintensities
