# Supplementary material for: Macular vessel density in the superficial plexus is not a proxy of cerebrovascular damage in non-demented individuals: data from the NORFACE cohort
Source: Alzheimers Res Ther. 2024 Feb 20;16:42. doi: 10.1186/s13195-024-01408-9 (PMC10877901; doi:10.1186/s13195-024-01408-9)
Supplement: Supplementary file 1 — Additional file 1. Multiple linear regression analyses of the association of clinical, demographic and biomarker variables with macular VD. Including hypertension, diabetes mellitus, dyslipidemia, heart disease, respiratory disease and smoking as adjusting factors. Significance was set up at p < 0.05. Abbreviations: A: amyloid; APOE: apolipoprotein E; CI: confidence interval; VD: vessel density. [file 13195_2024_1408_MOESM1_ESM.pdf]

Additional file 1

	Variables	Coefficient	95% CI	t	Significance	Beta
VD Nasal	Age	-0.10	-0.18 – -0.01	2.27	0.027*	-0.19
	Sex	-0.66	-1.82 – 0.51	1.11	0.267	-0.08
	Education	0.04	-0.08 – 0.16	0.66	0.514	0.05
	APOE ε4 status	0.19	-1.08 – 1.46	0.30	0.766	0.02
	A status	-0.55	-1.92 – 0.82	0.80	0.427	-0.07
	Syndromic diagnosis	1.03	-0.22 – 2.29	1.63	0.106	0.14
VD Temporal	Age	-0.10	-0.17 – -0.02	2.63	0.009*	-0.21
	Sex	-1.09	-2.08 – -0.09	2.15	0.033*	-0.15
	Education	0.02	-0.09 – 0.12	0.34	0.738	0.03
	APOE ε4 status	0.01	-1.08 – 1.09	0.01	0.991	0.00
	A status	0.04	-1.13 – 1.21	0.07	0.948	0.01
	Syndromic diagnosis	1.26	0.19 – 2.34	2.32	0.022*	0.18
VD Superior	Age	-0.09	-0.20 – 0.02	1.65	0.100	-0.14
	Sex	0.27	-1.16 – 1.71	0.37	0.709	0.03
	Education	0.14	-0.01 – 0.28	1.80	0.074	0.14
	APOE ε4 status	0.09	-1.48 – 1.66	0.11	0.910	0.01
	A status	-0.44	-2.13 – 1.25	0.52	0.606	-0.04
	Syndromic diagnosis	1.60	0.05 – 3.14	2.04	0.043*	0.17
VD Inferior	Age	-0.15	-0.28 – -0.03	2.43	0.016*	-0.21
	Sex	0.05	-1.62 – 1.71	0.05	0.957	0.00
	Education	0.05	-0.12 – 0.22	0.56	0.575	0.05
	APOE ε4 status	0.34	-1.48 – 2.16	0.37	0.712	0.03
	A status	0.18	-1.77 – 2.14	0.19	0.853	0.02
	Syndromic diagnosis	1.06	-0.73 – 2.85	1.16	0.246	0.10
