# Supplementary material for: Macular vessel density in the superficial plexus is not a proxy of cerebrovascular damage in non-demented individuals: data from the NORFACE cohort
Source: Alzheimers Res Ther. 2024 Feb 20;16:42. doi: 10.1186/s13195-024-01408-9 (PMC10877901; doi:10.1186/s13195-024-01408-9)
Supplement: Supplementary file 3 — Additional file 3. Multiple linear regression analysis of the association of regional macular VD with hippocampal volume without adjusting factors. Significance was set up at p < 0.0125. Abbreviation: VD: vessel density. [file 13195_2024_1408_MOESM3_ESM.pdf]

Additional file 3

Variables	Coefficient	t	Significance	Beta
VD Nasal	59.21	2.63	0.009*	0.24
VD Temporal	25.00	0.93	0.353	0.09
VD Superior	-19.01	1.19	0.238	-0.09
VD Inferior	30.66	2.46	0.015	0.18
