# Supplementary material for: Macular vessel density in the superficial plexus is not a proxy of cerebrovascular damage in non-demented individuals: data from the NORFACE cohort
Source: Alzheimers Res Ther. 2024 Feb 20;16:42. doi: 10.1186/s13195-024-01408-9 (PMC10877901; doi:10.1186/s13195-024-01408-9)
Supplement: Supplementary file 4 — Additional file 4. Multiple linear regression analysis of the association of regional macular VD with ventricle volume with adjusting factors. Including age, sex, syndromic diagnosis, hypertension, diabetes mellitus, dyslipidemia, heart disease, respiratory disease and smoking as adjusting factors. Significance was set up at p < 0.0125. Abbreviation: VD = vessel density. Note: A log transformation was applied to the ventricles volume measures. [file 13195_2024_1408_MOESM4_ESM.pdf]

Additional file 4

Variables	Coefficient	t	Significance	Beta
Age	0.02	4.78	<0.001*	0.37
Sex	0.11	1.55	0.122	0.11
Syndromic diagnosis	0.05	0.69	0.492	0.05
Hypertension	0.04	0.52	0.604	0.04
Diabetes mellitus	0.50	3.53	0.001*	0.24
Dyslipidemia	-0.03	0.37	0.711	-0.03
Heart disease	0.08	0.69	0.490	0.05
Respiratory disease	-0.00	0.04	0.968	-0.00
Smoking	-0.07	0.96	0.337	-0.07
VD Nasal	-0.00	0.04	0.969	-0.00
VD Temporal	-0.00	0.05	0.961	-0.00
VD Superior	-0.00	0.45	0.657	-0.04
VD Inferior	0.00	0.19	0.849	0.01
