# Supplementary material for: Macular vessel density in the superficial plexus is not a proxy of cerebrovascular damage in non-demented individuals: data from the NORFACE cohort
Source: Alzheimers Res Ther. 2024 Feb 20;16:42. doi: 10.1186/s13195-024-01408-9 (PMC10877901; doi:10.1186/s13195-024-01408-9)
Supplement: Supplementary file 5 — Additional file 5: Multiple linear regression analysis of the association of regional macular VD with ventricles volume without adjusting factors. Significance was set up at p < 0.0125. Abbreviation: VD = vessel density. Note: A log transformation was applied to the ventricles volume measures. [file 13195_2024_1408_MOESM5_ESM.pdf]

Additional file 5

Variables	Coefficient	t	Significance	Beta
VD Nasal	-0.00	0.32	0.752	-0.03
VD Temporal	-0.02	1.05	0.295	-0.11
VD Superior	-0.00	0.19	0.849	-0.02
VD Inferior	-0.01	0.92	0.361	-0.07
