# Supplementary material for: Macular vessel density in the superficial plexus is not a proxy of cerebrovascular damage in non-demented individuals: data from the NORFACE cohort
Source: Alzheimers Res Ther. 2024 Feb 20;16:42. doi: 10.1186/s13195-024-01408-9 (PMC10877901; doi:10.1186/s13195-024-01408-9)
Supplement: Supplementary file 7 — Additional file 7. Multiple linear regression analysis of the association of regional macular VD with ADCS thickness without adjusting factors. Significance was set up at p < 0.0125. Abbreviations: ADCS: Alzheimer´s disease cortical signature; VD = vessel density. [file 13195_2024_1408_MOESM7_ESM.pdf]

Additional file 7

Variables	Coefficient	t	Significance	Beta
VD Nasal	0.00	0.94	0.347	0.09
VD Temporal	-0.00	0.71	0.481	-0.07
VD Superior	-0.00	0.71	0.481	-0.06
VD Inferior	-0.00	1.59	0.115	-0.12
