# Supplementary material for: Macular vessel density in the superficial plexus is not a proxy of cerebrovascular damage in non-demented individuals: data from the NORFACE cohort
Source: Alzheimers Res Ther. 2024 Feb 20;16:42. doi: 10.1186/s13195-024-01408-9 (PMC10877901; doi:10.1186/s13195-024-01408-9)
Supplement: Supplementary file 8 — Additional file 8. Logistic regression analysis of the interaction of the A status and macular VD in discriminating Fazekas categories. Including age, sex, syndromic diagnosis, hypertension, diabetes mellitus, dyslipidemia, heart disease, respiratory disease and smoking as adjusting factors. Significance was set up at p < 0.0125. Abbreviations: CI: confidence interval; OR: odds ratio; VD: vessel density. [file 13195_2024_1408_MOESM8_ESM.pdf]

Additional file 8

Variables	OR				95 CI%				Significance			
Age	1.14	1.14	1.15	1.15	1.04 – 1.27	1.04 –1.27	1.05 –1.28	1.05 –1.28	0.011*	0.010*	0.006*	0.004*
Sex	2.47	2.73	2.26	1.79	0.70 –10.73	0.76 –12.14	0.66 –9.15	0.51 –7.35	0.186	0.147	0.216	0.384
Syndromic diagnosis	0.44	0.41	0.43	0.41	0.11 – 1.64	0.10 –1.50	0.10 –1.61	0.10 –1.53	0.237	0.193	0.224	0.200
Hypertension	6.04	5.59	5.92	6.05	1.82 –22.71	1.65 –21.17	1.77 –22.47	1.80 –23.11	0.005*	0.007*	0.005*	0.005*
Diabetes mellitus	1.38	1.45	1.27	1.51	0.17 – 7.71	0.17 – 8.18	0.15 –7.09	0.18 –8.79	0.735	0.696	0.798	0.668
Dyslipidemia	0.38	0.36	0.37	0.39	0.10 – 1.28	0.10 –1.23	0.10 –1.26	0.10 –1.36	0.135	0.120	0.128	0.158
Heart disease	0.78	0.80	0.76	0.72	0.08 – 4.87	0.08 –5.32	0.08 –4.58	0.08 –4.29	0.805	0.832	0.787	0.740
Respiratory disease	4.22	4.17	4.17	4.09	0.80 –22.05	0.79 –21.98	0.79 –21.97	0.73 –22.82	0.081	0.085	0.086	0.101
Smoking	6.57	7.75	6.82	5.95	1.76 –29.29	1.98 –37.72	1.82 –30.38	1.51 –27.37	0.008*	0.006*	0.007*	0.014
A status	0.00	0.00	7.02	inf	0.00 – inf	0.00 –68.42	0.00 - inf	0.46 - inf	0.476	0.152	0.743	0.085
VD Nasal	1.01	1.03	1.04	1.06	0.80 – 1.26	0.83 –1.28	0.85 –1.29	0.87 –1.32	0.950	0.805	0.707	0.546
VD Temporal	1.17	1.08	1.18	1.12	0.91 – 1.53	0.81 –1.45	0.92 –1.54	0.88 –1.47	0.241	0.605	0.203	0.389
VD Superior	0.91	0.91	0.92	0.90	0.80 – 1.02	0.80 –1.02	0.80 –1.08	0.79 –1.01	0.115	0.110	0.293	0.088
VD Inferior	1.03	1.03	1.04	1.20	0.90 – 1.15	0.91 –1.15	0.92 –1.15	0.99 –1.50	0.665	0.626	0.514	0.080
VD Nasal * A status	1.17				0.78 – 1.91				0.487			
VD Temporal * A status		1.34				0.91 –2.05				0.155		
VD Superior * A status			0.95				0.71 –1.19				0.709	
VD Inferior * A status				0.80				0.61 –1.01				0.079
