# Supplementary material for: Macular vessel density in the superficial plexus is not a proxy of cerebrovascular damage in non-demented individuals: data from the NORFACE cohort
Source: Alzheimers Res Ther. 2024 Feb 20;16:42. doi: 10.1186/s13195-024-01408-9 (PMC10877901; doi:10.1186/s13195-024-01408-9)
Supplement: Supplementary file 9 — Additional file 9. Multivariate regression analysis of the interaction of the A status and macular VD in discriminating WMH volume. Including age, sex, syndromic diagnosis, hypertension, diabetes mellitus, dyslipidemia, heart disease, respiratory disease and smoking as adjusting factors. Significance was set up at p < 0.0125. Abbreviations: A: amyloid; V: vessel density. [file 13195_2024_1408_MOESM9_ESM.pdf]

Additional file 9

Variables	Coefficient				Significance				Beta			
Age	0.05	0.05	0.05	0.05	<0.001*	<0.001*	<0.001*	<0.001*	0.38	0.38	0.38	0.38
Sex	0.06	0.06	0.05	0.05	0.680	0.692	0.704	0.748	0.03	0.03	0.03	0.02
Syndromic diagnosis	0.21	0.21	0.20	0.21	0.149	0.146	0.161	0.154	0.11	0.11	0.10	0.11
Hypertension	0.33	0.33	0.33	0.33	0.032	0.032	0.032	0.031	0.16	0.16	0.16	0.16
Diabetes mellitus	0.59	0.59	0.59	0.60	0.042	0.043	0.042	0.038	0.14	0.14	0.14	0.14
Dyslipidemia	-0.05	-0.05	-0.05	-0.03	0.749	0.714	0.740	0.843	-0.02	-0.03	-0.02	-0.01
Heart disease	0.09	0.10	0.09	0.07	0.683	0.674	0.711	0.751	0.03	0.03	0.03	0.02
Respiratory disease	0.37	0.36	0.37	0.37	0.095	0.104	0.091	0.095	0.12	0.11	0.12	0.12
Smoking	-0.04	-0.04	-0.03	-0.05	0.801	0.809	0.824	0.749	-0.02	-0.02	-0.02	-0.02
A status	-0.71	-1.28	0.03	1.00	0.691	0.493	0.982	0.417	-0.33	-0.59	0.02	0.46
VD Nasal	-0.05	-0.05	-0.05	-0.04	0.063	0.043	0.048	0.077	-0.19	-0.18	-0.17	-0.16
VD Temporal	0.05	0.05	0.06	0.05	0.058	0.152	0.053	0.071	0.19	0.16	0.19	0.18
VD Superior	-0.01	-0.01	-0.01	-0.01	0.550	0.549	0.650	0.484	-0.05	-0.05	-0.04	-0.06
VD Inferior	0.01	0.01	0.01	0.02	0.694	0.676	0.633	0.417	0.03	0.03	0.03	0.11
VD Nasal * A status	0.01				0.726				0.29			
VD Temporal * A status		0.03				0.520				0.55		
VD Superior * A status			-0.00				0.933				-0.05	
VD Inferior * A status				-0.02				0.374				-0.51
