# Supplementary material for: Macular vessel density in the superficial plexus is not a proxy of cerebrovascular damage in non-demented individuals: data from the NORFACE cohort
Source: Alzheimers Res Ther. 2024 Feb 20;16:42. doi: 10.1186/s13195-024-01408-9 (PMC10877901; doi:10.1186/s13195-024-01408-9)
Supplement: Supplementary file 10 — Additional file 10. Multivariate regression analysis of the interaction of the A status and macular VD in discriminating hippocampal volume. Including age, sex, syndromic diagnosis, hypertension, diabetes mellitus, dyslipidemia, heart disease, respiratory disease and smoking as adjusting factors. Significance was set up at p < 0.0125. Abbreviations: A: amyloid; VD: vessel density. [file 13195_2024_1408_MOESM10_ESM.pdf]

Additional file 10

Variables	Coefficient				Significance				Beta			
Age	-63.56	-62.60	-62.48	-62.10	<0.001*	<0.001*	<0.001*	<0.001*	-0.49	-0.48	-0.48	-0.48
Sex	-139.25	-151.30	-156.84	-156.08	0.259	0.219	0.203	0.205	-0.07	-0.08	-0.08	-0.08
Syndromic diagnosis	107.98	87.33	80.88	83.69	0.403	0.492	0.523	0.509	0.06	0.05	0.04	0.04
Hypertension	-118.03	-115.45	-110.62	-114.93	0.373	0.385	0.406	0.387	-0.06	-0.06	-0.05	-0.06
Diabetes mellitus	-10.07	-9.75	-3.45	-3.09	0.968	0.969	0.989	0.990	-0.00	-0.00	-0.00	-0.00
Dyslipidemia	-140.97	-148.35	-146.81	-138.03	0.253	0.231	0.235	0.270	-0.07	-0.08	-0.08	-0.07
Heart disease	-73.65	-83.81	-81.87	-95.20	0.711	0.674	0.682	0.633	-0.02	-0.03	-0.03	-0.03
Respiratory disease	-177.78	-175.75	-175.14	-169.53	0.350	0.358	0.360	0.373	-0.06	-0.06	-0.06	-0.06
Smoking	68.75	77.08	76.18	72.89	0.594	0.550	0.555	0.574	0.03	0.04	0.04	0.04
A status	-inf	-758.85	-517.60	394.04	0.319	0.639	0.673	0.714	-0.74	-0.36	-0.25	0.19
VD Nasal	41.33	52.75	53.35	55.15	0.078	0.008*	0.008*	0.007*	0.17	0.21	0.21	0.22
VD Temporal	-17.20	-21.03	-15.49	-16.60	0.487	0.449	0.531	0.506	-0.06	-0.08	-0.06	-0.06
VD Superior	-11.97	-12.03	-15.80	-12.71	0.403	0.401	0.358	0.379	-0.06	-0.06	-0.08	-0.06
VD Inferior	10.19	12.11	12.63	18.26	0.375	0.284	0.261	0.313	0.06	0.07	0.07	0.10
VD Nasal * A status	32.17				0.328				0.72			
VD Temporal * A status		15.86				0.653				0.35		
VD Superior * A status			9.99				0.691				0.23	
VD Inferior * A status				-9.00				0.688				-0.21
