# Supplementary material for: Macular vessel density in the superficial plexus is not a proxy of cerebrovascular damage in non-demented individuals: data from the NORFACE cohort
Source: Alzheimers Res Ther. 2024 Feb 20;16:42. doi: 10.1186/s13195-024-01408-9 (PMC10877901; doi:10.1186/s13195-024-01408-9)
Supplement: Supplementary file 12 — Additional file 12. Multivariate regression analysis of the interaction of the A status and macular VD in discriminating ACDS thickness. Including age, sex, syndromic diagnosis, hypertension, diabetes mellitus, dyslipidemia, heart disease, respiratory disease and smoking as adjusting factors. Significance was set up at p < 0.0125. Abbreviations: A: amyloid; ADCS: Alzheimer´s disease cortical signature; VD: vessel density. [file 13195_2024_1408_MOESM12_ESM.pdf]

Additional file 12

Variables	Coefficient				Significance				Beta			
Age	-0.00	-0.00	-0.00	-0.00	0.624	0.672	0.572	0.698	-0.04	-0.03	-0.04	-0.03
Sex	0.01	0.01	0.01	0.01	0.500	0.540	0.640	0.574	0.05	0.04	0.03	0.04
Syndromic diagnosis	-0.15	-0.15	-0.15	-0.15	<0.001*	<0.001*	<0.001*	<0.001*	-0.48	-0.49	-0.49	-0.49
Hypertension	-0.02	-0.02	-0.02	-0.02	0.349	0.356	0.428	0.355	-0.07	-0.06	-0.06	-0.07
Diabetes mellitus	-0.01	-0.01	-0.00	-0.00	0.887	0.888	0.945	0.915	-0.01	-0.01	-0.00	-0.01
Dyslipidemia	-0.05	-0.05	-0.05	-0.05	0.023	0.021	0.018	0.029	-0.16	-0.16	-0.16	-0.15
Heart disease	0.03	0.03	0.03	0.03	0.405	0.425	0.335	0.465	0.06	0.05	0.06	0.05
Respiratory disease	0.02	0.02	0.01	0.02	0.576	0.572	0.682	0.564	0.04	0.04	0.03	0.04
Smoking	-0.03	-0.03	-0.03	-0.03	0.135	0.146	0.123	0.130	-0.10	-0.10	-0.11	-0.11
A status	-0.19	-0.09	-0.40	0.11	0.488	0.743	0.056	0.543	-0.55	-0.27	-1.18	0.33
VD Nasal	0.00	0.00	0.00	0.00	0.537	0.270	0.275	0.212	0.06	0.09	0.09	0.11
VD Temporal	0.00	0.00	0.00	0.00	0.702	0.795	0.683	0.733	0.04	0.03	0.04	0.03
VD Superior	-0.00	-0.00	-0.00	-0.00	0.490	0.489	0.109	0.442	-0.05	-0.05	-0.15	-0.06
VD Inferior	-0.00	-0.00	-0.00	-0.00	0.281	0.325	0.341	0.956	-0.08	-0.07	-0.06	-0.01
VD Nasal * A status	0.00				0.523				0.50			
VD Temporal * A status		0.00				0.785				0.22		
VD Superior * A status			0.01				0.065				1.14	
VD Inferior * A status				-0.00				0.486				-0.38
